# College and Hospital Notes

**Published:** 1905-04

**Authors:** 


### COLLEGE AND HOSPITAL NOTES.

THE University of Buffalo will hold its fifty-ninth annual commencement at the Teck Theater, Thursday, May 4, 1905, at 11 o'clock a. m. The address to the graduates will be delivered by the Reverend Richard Earle Locke, of Buffalo, and other ceremonies will be observed after the usual form.

The Alumni Association of the Medical Department will observe its anniversary May 3 and 4. Wednesday, May 3, will be given over to clinics, and on Thursday the usual class reunions and other ceremonies will take place. The annual banquet will be given at the Hotel Iroquois, Thursday evening, May 4. Dr. DeLancey Rochester is the president, and Dr. Albert T. Lytle is the chairman of the executive committee.

---

DR. L. DUNCAN BULKLEY, announces that at the close of his special course of lectures at the New York Skin and Cancer Hospital, 19th street and Second avenue, New York, on Wednesday, March 29, 1905, at 4.15 p. m., there will be a clinic on cancer by Dr. William Seaman Bainbridge, of the hospital staff. A number of interesting cases will be on exhibition.

